# Entropy-Based TOA Estimation and SVM-Based Ranging Error Mitigation in UWB Ranging Systems

**DOI:** 10.3390/s150511701

**Published:** 2015-05-21

**Authors:** Zhendong Yin, Kai Cui, Zhilu Wu, Liang Yin

**Affiliations:** School of Electronics and Information Engineering, Harbin Institute of Technology, Harbin 150001, China; E-Mails: yinzhendong@hit.edu.cn (Z.Y.); wuzhilu@hit.edu.cn (Z.W.); ylxt2009@163.com (L.Y.)

**Keywords:** ranging, entropy, TOA, UWB, error mitigation, SVM

## Abstract

The major challenges for Ultra-wide Band (UWB) indoor ranging systems are the dense multipath and non-line-of-sight (NLOS) problems of the indoor environment. To precisely estimate the time of arrival (TOA) of the first path (FP) in such a poor environment, a novel approach of entropy-based TOA estimation and support vector machine (SVM) regression-based ranging error mitigation is proposed in this paper. The proposed method can estimate the TOA precisely by measuring the randomness of the received signals and mitigate the ranging error without the recognition of the channel conditions. The entropy is used to measure the randomness of the received signals and the FP can be determined by the decision of the sample which is followed by a great entropy decrease. The SVM regression is employed to perform the ranging-error mitigation by the modeling of the regressor between the characteristics of received signals and the ranging error. The presented numerical simulation results show that the proposed approach achieves significant performance improvements in the CM1 to CM4 channels of the IEEE 802.15.4a standard, as compared to conventional approaches.

## 1. Introduction

Navigating in indoor environments beyond the coverage of GPS, locating people or objects in particular areas and locating nodes in wireless sensor networks are applicable in both industry and everyday life [1,2,3]. Ultra-wide band (UWB) transmission [4,5,6] is a promising technology for ranging and localization in poor indoor environments and accuracy-critical applications [7,8], owing to its large bandwidth, high time-delay resolution [9] and good obstacle-penetration capability [10]. A time-based ranging method such as time of arrival (TOA) is one of the best techniques for UWB ranging [11], as it can take full advantage of the large bandwidth and time-delay resolution of the UWB technique [12,13,14]. The key to the TOA ranging method is to detect the first path (FP) and determine its arrival time. The accuracy of TOA estimation is thus highly dependent on the quality of FP detection [15,16].

However, FP detection is always faced with a number of technical challenges, including interference from other wireless systems, dense multipath effects, and the existence of both line-of-sight (LOS) and non-line-of-sight (NLOS) conditions [17,18]. The dense multipath effects and NLOS conditions are critical for high-resolution ranging and localization systems since they cause biased FP detection, which severely degrades ranging and localization performance [19]. Therefore, it is necessary to understand the factors that cause degraded ranging accuracy, and to develop techniques that can estimate the TOA precisely so as to avoid or mitigate their impacts.

**Figure 1:**
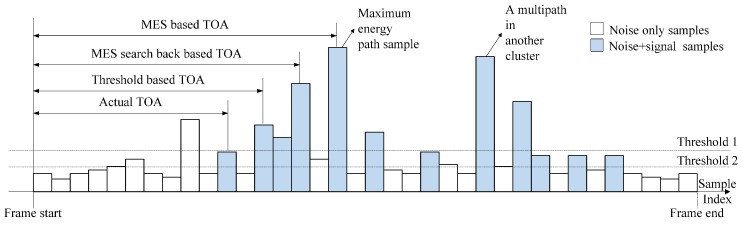
Illustration of conventional TOA approaches.

Many approaches have been proposed to address these problems. For accuracy improvements, most of the approaches focus on modifications of the FP detection algorithms, such as maximum energy selection (MES), MES searching-back (MES-SB), coherent detection and some threshold-crossing-based methods [20,21,22,23,24], as shown in Figure 1. Other approaches try to build mathematical models for range information [25], which, however, turns out to be complicated and relies too much on prior information. As for the MES methods, the energy block with the maximal energy is considered as the one containing the FP, but the position of the FP in the energy block cannot be determined, and under NLOS conditions, there is a delay between the energy block that contains the FP and the maximal energy block, which results in a biased estimation. There have been some achievements in the MES-SB method, but the in-block problem remains. In the case of threshold-crossing-based methods, the sample or the energy block which first exceeds the threshold is taken as the FP, so it is very important to select the appropriate threshold. However, in NLOS conditions, there is a delay between the FP and the strongest path (SP), and sometimes the FP is even lower than the noise, so it is hard to detect the FP with conventional threshold-crossing. A number of modified algorithms are proposed to determine the threshold, but many of them are parametric and complex [26,27], which requires prior information or complicated calculations, and the estimation results are still far from optimal. Therefore, a method of which the threshold is not so strictly required and the accuracy is acceptable is worth consideration.

As to the existence of both the LOS and the NLOS conditions, the approaches proposed in the literature can be roughly classified as NLOS identification and NLOS mitigation [28]. In NLOS identification, the goal is to detect the existence of a NLOS condition between a transmitter and a receiver [29]. It can be performed by analyzing the received signals, or analyzing the variance of range estimates from a single source [30,31]. In NLOS mitigation, the goal is to reduce the effect of the ranging errors in NLOS conditions [32,33,34,35]. This can be achieved by direct path detection which eliminates the ranging results under NLOS conditions to improve the performance. Statistics-based mitigation, which requires prior statistical characteristics of received signals under NLOS conditions, is also used in the literature [36]. NLOS mitigation can be combined with NLOS identification by assigning different weights to LOS and NLOS signals [37], but the selection of weights is complicated, so a method which can mitigate the ranging error without recognizing channel conditions is worth consideration.

In this paper, a novel approach of entropy-based TOA estimation and SVM-based ranging error mitigation is proposed on the basis of our research results, which can estimate the TOA more precisely by measuring the randomness of the received signals and mitigate the ranging error without recognizing channel conditions. Through a calculation of the entropy of the received signals, the FP can be better detected and the TOA can be estimated more accurately. Through a regression with SVM, the regression model between the ranging error and the characteristics of received signals which can perform the error mitigation is built. The approach proposed in this paper is validated with the IEEE 802.15.4a standard and the simulation results have proved its effectiveness and robustness.

The main body of the paper is organized as follows: In Section 2, a description of TOA-based UWB ranging models, the set of factors that degrade the ranging accuracy and the problem statement are given. In Section 3 background information is provided for the entropy-based TOA estimation and detailed steps of the entropy calculation. Section 4 comes up with a description of the theory of support vector regression and the detailed mitigation procedure. The corresponding performance evaluation and the simulation results as well as analysis are discussed respectively at the end of Section 3 and Section 4. Finally, conclusions are given in Section 5.

## 2. Problem Statement

### 2.1. UWB Ranging System Models

First, the signal waveform of the UWB ranging systems provides a starting point. The Gaussian pulse and its derivatives in different orders are often used as UWB transmitted signals. Owing to the simplicity of transmission and realization, the second order derivatives of the Gaussian pulses are used as UWB signals, which can be expressed as Equation (1):

(1) $$s(t)=\frac{{\text{d}}^{2}p(t)}{\text{d}t^{2}}=(1-4\pi \frac{t^{2}}{\alpha^{2}})e^{\frac{-2\pi t^{2}}{\alpha^{2}}}$$

where *p*(*t*) is the standard Gaussian function. $\alpha^{2}=4\pi \sigma^{2}$ is called the shape factor of the waveform.

Then, the channel models of the UWB ranging systems are to be considered. Dense multipath and the existence of both LOS and NLOS conditions are the most important characteristics of the channel model for indoor UWB ranging systems. The IEEE 802.15.4a standard is the first international standard that specifies a wireless physical layer to ensure precise ranging. It should allow for high aggregate throughput communications with a precise ranging capability.

IEEE802.15.4a contains four different models: (1) an UWB model, with the frequency range spanning from 2 to 10 GHz; (2) an UWB model for the frequency range from 100 to 1000 MHz; (3) a narrowband model for the frequency range around 1 MHz; (4) a body-area network range from 2 to 10 GHz. In consideration of LOS and NLOS conditions and application scene, the IEEE 802.15.4a standard can be divided into eight categories, as shown in Table 1.

**Table 1:** IEEE802.15.4a channel models.

Number of Channel Model	Channel Model Description
CM1	LOS of indoor residential (7~20 m)
CM2	NLOS of indoor residential (7~20 m)
CM3	LOS of indoor office (3~28 m)
CM4	NLOS of indoor office (3~28 m)
CM5	LOS of outdoor (5~17 m)
CM6	NLOS of outdoor (5~17 m)
CM7	LOS of industrial (2~8 m)
CM8	NLOS of industrial (2~8 m)

In consideration of the application environment in the research, an UWB model for the frequency range from 2 to 10 GHz and channel models CM1 to CM4 are used to simulate and validate the approach proposed in this paper. As paths generally arrive in clusters, the IEEE802.15.4a standard is presented to modify the classical Saleh-Valenzuela model, and the channel impulse response can be expressed as Equation (2):

(2) $$h(t)=\sum_{l=0}^{L}\sum_{k=0}^{K}\alpha_{k,l}\exp(j\phi_{k,l})\delta (t-T_{l}-\tau_{k,l})$$

where $\left\{\alpha_{k,l}\right\}$ is the gain factor of each multipath, *T_l_* denotes the time delay of the *l*th cluster, $\tau_{k,l}$ denotes the time delay of the *k*th multipath component in the *l*th cluster, phase $\phi_{k\text{,}l}$ is distributed uniformly in [0,2π), *L* denotes the number of clusters.

Then the signal received can be expressed as Equation (3):

(3) r(t)=s(t)*h(t)+n(t)

where *n*(*t*) denotes additive white Gaussian noise, *s*(*t*) is the UWB signal as is expressed in Equation (1). Then, the signals received can be expressed as Equation (4):

(4) $$r(t)=\sum_{l\text{=}0}^{L}\sum_{k\text{=}0}^{K}\alpha_{k\text{,}l}\exp\text{(}j\phi_{k\text{,}l}\text{)}s\text{(}t-T_{l}-\tau_{k\text{,}l}\text{)}+n(t)$$

The characteristics of channels can be summarized as follows: these channels are all dense multipath ones with severe multipath effects. The paths arrive in clusters. In the LOS channel, the first path and the strongest path almost arrive at the same time, so the FP can be easily detected, but in the NLOS channel, there is a time-delay between the first path and the strongest path, which brings forth a number of challenges to FP detection.

### 2.2. Ranging Error Analysis

The TOA-based ranging method is among the best techniques for UWB ranging, because it can make full use of the large bandwidth and fine space resolution of the UWB technique. TOA can be defined as the estimation of the time delay between transmitted signals and received signals. Perfect synchronization between the transmitter and the receiver is taken for granted here, which is a common assumption in other literature resources. If the time delay is ΔT, the estimated distance can be expressed as d=ΔT×c, where *c* is velocity of electromagnetic wave. The key of TOA estimation is the detection of the first path and the determination of its arrival time.

According to the literature [9], the Cramer-Rao Lower Bound (CRLB) of distance estimation using the TOA method in UWB ranging systems can be expressed as Equation (5):

(5) $$V\{\hat{d}\}\geq \sqrt{\frac{c^{2}}{8\pi^{2}\beta^{2}SNR}}$$

where $\hat{d}$ is the distance estimation, *β* is the bandwidth of the UWB signal and *SNR* is the signal-to-noise ratio, so the theoretically best achievable accuracy of range estimation is centimeter in size, which can be derived from Equation (5), but in simulations or practical applications, the ranging errors are often tens of centimeters or even more than 1 m, which goes far beyond the theoretically achievable accuracy. This problem is mainly caused by the errors in FP detection accuracy, which is subject to the dense multipath and NLOS conditions.

In consideration of these issues, an entropy-based TOA estimation and SVM regression-based error mitigation method are proposed. The drastic entropy decrease reflects the essential difference of noise and multipath signals which is helpful to more accurately determine the arrival time of FP, and the SVM regression with characteristics of received signals can mitigate the ranging error in a low-complexity way. This signifies a departure from conventional ranging and mitigating approaches and leads to performance improvements as well as reduction in complexity. The details will be investigated in the next two sections.

## 3. Entropy-Based TOA Estimation in UWB Ranging

### 3.1. The Theory of Entropy

In this section, a brief description is given of the principles of entropy. Let ***x*** be a random variable with a probability mass function $P(x_{i})$. The entropy is defined as:

(6) $$H(x)=-\sum_{i}P(x_{i})\log_{b}P(x_{i})$$

where *b* is the base of the logarithm used. Common values of *b* are 2, Euler’s number *e*, and 10, and the unit of entropy is shannon for *b* = 2, nat for *b* = e, and hartley for *b* = 10.

Entropy is a measurement of the unpredictability of a system or a random process. The larger the entropy is, the more random the data are. In consideration of the randomness of noise-only-samples and the relative stableness of signals samples, entropy was used in our research to measure the randomness of received signals to detect the first path.

As mentioned above, the detection accuracy of FP in conventional approaches degrades the TOA estimation performance, especially in the TC method. Because of the existence of dense multipath and NLOS conditions, the amplitude of the FP may be very small and sometimes even smaller than the noise. If a bad threshold is set under this condition, an accurate detection of the FP will be impossible, but with the information entropy theory, by which the degree of disorder and randomness become measurable, this problem can be considered from another angle. Noise samples can cross the threshold if the threshold is lower than the optimal one, as the threshold 2 set in Figure 1, but those TC-noise samples occur randomly in the noise region. It is an event of small probability that the indices of two TC-noise samples are the same. If the indices of those TC noise samples can be viewed as a random variable, it is very random. The entropy is a good parameter to measure its randomness. As the occurrences of TC noise samples are highly random, their corresponding entropy is high. On the contrary, the signal samples including FP are relative definite, even with noise. They can exceed the threshold in most cases and the corresponding entropy is low. As a result, the FP can be determined by choosing the sample which is followed by a great entropy decrease.

### 3.2. Proposed Entropy Based Method

**Figure 2:**
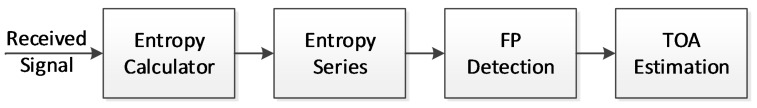
Procedure of entropy-based TOA estimation.

The flow chart of the proposed method is illustrated in Figure 2 and it is described in details by the following procedure. It can be assumed that the channel is the time invariant during the whole observation time, so the FP are fixed; whereas the noise is random and noises in different frames are statistically independent and random.

First, setting of a threshold is required in the entropy-based approach. The threshold is chosen to make sure that the FP and other signal samples exceed the threshold in most cases, but the noise samples can exceed the threshold randomly. As IEEE 802.15.4a channel models are complex, the corresponding additive white Gaussian noise is also complex and can be expressed as X+Yi, assuming that X,Y are independently and identically Gaussian-distributed with equal variance and zero mean as $\left(0,\sigma^{2}\right)$. Then, its magnitude $Z=\sqrt{X^{2}+Y^{2}}$ can be characterized by a Rayleigh distribution. The probability density function (PDF) of a Rayleigh distribution can be expressed as:

(7) $$f\left(Z;\sigma \right)=\frac{Z}{\sigma^{2}}e^{-\;\frac{Z^{2}}{2\sigma^{2}}},Z\in [0,+\infty )$$

The cumulative distribution function (CDF) of a Rayleigh distribution can be expressed as:

(8) $$\Phi \left(Z\right)=1-e^{-\;\frac{Z^{2}}{2\sigma^{2}}},Z\in [0,+\infty )$$

Then, based on the signal detection theory, we can get the equation:

(9) $$p_{fa}=1-\Phi \left(\eta \right)=1-(1-e^{-\;\frac{\eta^{2}}{2\sigma^{2}}})=e^{-\;\frac{\eta^{2}}{2\sigma^{2}}}$$

where η is the threshold, $p_{fa}$ is the false alarm rate.

Then threshold can be derived from Equation (9):

(10) $$\eta =\sqrt{-2\ln\left(p_{fa}\right)}\sigma$$

Let $\sqrt{-2\ln\left(p_{fa}\right)}=a$, then we can get:

(11) η=aσ

where *a* is called threshold factor. If a is a constant factor, it means a constant false alarm rate (CFAR) condition. As the entropy-based methods have a looser demand on the threshold and the true value of σ is unknown, σ can be replaced by the standard deviation of received noise which can be obtained from the guard interval of received signals, and then, only the threshold factor *a* is undetermined.

In order to select the proper threshold factor, a numerical simulation was done to show the ranging performance under different thresholds. The threshold factor *a* ranges from 0 to 10, the SNR ranges from −10 dB to 15 dB with the step of 5 dB. IEEE 802.15.4a CM1-CM4 are used as channel models and root-mean-squared error (RMSE) is used to evaluate the quality of the ranging result under different thresholds with various SNRs, as shown in Equation (12):

(12) $$RMSE=\sqrt{\sum_{k=1}^{K_{S}}{\left(\left(\tau_{k}-\tau_{toa}\right)*c\right)}^{2}/K_{S}}$$

where $\tau_{k}$ is the TOA estimation of each realization, and $\tau_{toa}$ is the actual TOA of each realization, *c* is velocity of the electromagnetic wave and $K_{S}$ is the times of simulation, $K_{S}$ is set as 50 this time.

**Figure 3:**
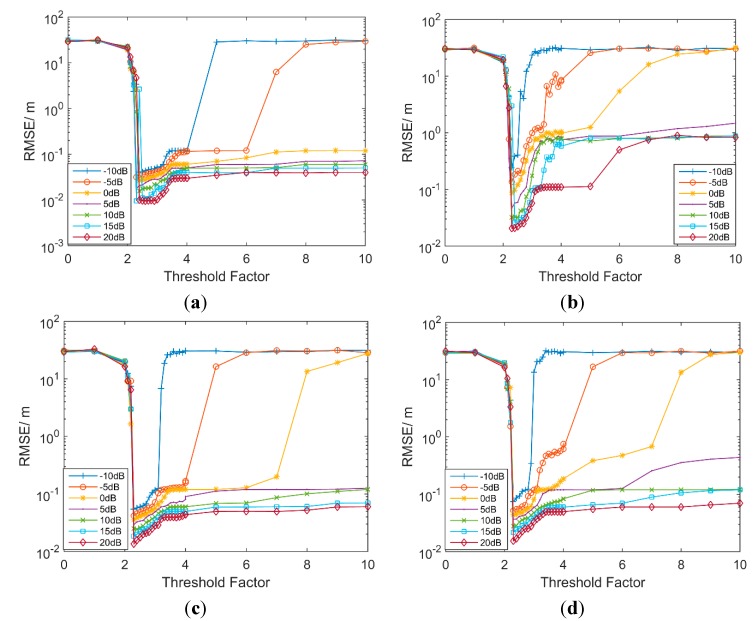
RMSE as a function of the threshold factor in IEEE802.15.4a. (**a**) CM1; (**b**) CM2; (**c**) CM3; (**d**) CM4.

According to the simulation results in Figure 3, it can be seen that the RMSE decreases significantly at the beginning under all conditions. After reaching the lowest point at about 2.5, it starts to increase and the rate of growth depends on SNRs. As seen in Figure 3, RMSE is relatively low over a wide threshold range for high SNRs under LOS conditions and a little bit tight for low SNRs or NLOS conditions, proving that the entropy-based method has a looser demand for the threshold. When the threshold factor is about 2.5, the RMSE is at the lowest point, so the threshold factor *a* can be set as 2.5. Therefore, a proper threshold can be obtained in a simple way. In consideration of the rationality of the layout, a detailed analysis of the connection between threshold factor and the ranging error and why 2.5 is the best threshold factor would be provided in the Appendix.

Then, the received signals can be expressed as R, the size of R is *N* by *K*, where *N* is the number of frames, *K* is the length of received signals, $R_{n,k}$ means *k*th sample in the *nth* frame. By comparing $R_{n,k}$ with the threshold η, a new matrix U can be obtained in accordance with the Equation (13), U has the same size as R and the elements in U can be expressed as:

(13) $$U_{n,k}=\{\begin{array}{cc} 1 & if\;R_{n,k}>\eta \\ 0 & \;else \end{array}\;,\;n=1,\ldots ,N,k=1,\ldots ,K$$

Next, as is mentioned above, those TC noise samples occur randomly in the noise region due to their randomness. It is an event of small probability that the indices of two TC noise samples are the same. Therefore, for the *k*th index, the distribution of the last TC sample before the *k*th index among all frames is examined in the new sequence U. For *k*th sample, a subset can be obtained as expressed in Equation (14):

(14) $$\Phi_{\text{k}}=\left\{l_{n}\right\},l_{n}=\underset{m}{\arg}\;\max\;m\;s.t.U_{n,m}=1\;and\;m<k,\;n=1,\ldots ,N$$

As a result, *K* subsets $\Phi_{\text{k}}$ can be obtained corresponding to *K* indices of received signals. Next, the entropy of $\Phi_{\text{k}}$ can be calculated by Equation (15):

(15) $$E_{k}=-\sum_{i=1}^{I}\left(p_{i}/P\right)\log_{b}\left(p_{i}/P\right)$$

where *P* is the total number of $\Phi_{\text{k}}$, the number of non-repetitive elements is *I*, $s_{i}$ is an element of $\Phi_{\text{k}}$ and the frequency of occurrence of $s_{i}$ in the set is $p_{\text{i}}$. Finally entropy series ***E*** can be obtained. As is known in information theory, a bigger entropy will be achieved when all non-repetitive elements occur with the same frequency, which means that the set is randomly distributed, like the noise regions in received signals. As to the signal region, the corresponding entropies are relatively low, because these samples can exceed the threshold in most cases.

Finally, in consideration of the simplicity and easy to implement, the turning point of entropy series is determined as the arrival index of FP. The turning point can be obtained as follows. Firstly, a differential entropy series can be obtained by Equation (16):

(16) $$E_{k}=E_{k+1}-E_{k},k=1,\ldots ,K$$

The index of maximal differential entropy can be obtained by Equation (17):

(17) $$n_{\max}=\arg\underset{k}{\max}\left|E_{k}\right|,k=1,\ldots ,K-1$$

The index of maximal differential entropy always delays, compared to the index of turning point. If we want to obtain the index of the turning point, a search back is to be performed as expressed in Equation (18):

(18) $$n_{\max}=\{\begin{array}{ll} n_{\max} & \;if\;E_{n_{\max}}\geq E_{n_{\max}-1}\\ n_{\max}-1 & \;if\;E_{n_{\max}}<E_{n_{\max}-1} \end{array}$$

After the search back, let *n_max_* = *n_toa_*, and the arrival time of FP can be determined by:

(19) $$t_{toa}=n_{toa}T_{sample}-\tau_{guard}$$

where $T_{sample}$ is the sample time and $\tau_{guard}$ is the guard interval.

**Figure 4:**
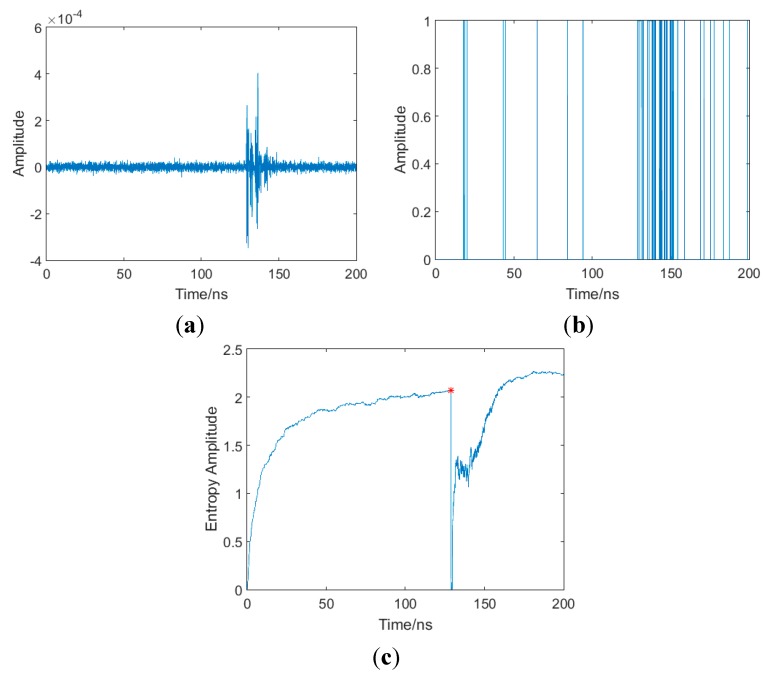
Illustration of the procedure in one realization (CM1 channel, SNR = 10 dB). (**a**) Received signal; (**b**) threshold crossing of nth sample; (**c**) entropy of received signal.

Figure 4 is an illustration of the whole procedure in one realization, in which the CM1 channel, *SNR* = 10 dB is set as an example, and the results under other conditions are similar. From Figure 4a, it can be seen that the received signal is in cluster due to the multipath effects. As shown in Figure 4b, samples in the signal region can cross threshold in most cases, while other noise samples occasionally exceed the threshold. The entropy series ***E*** can be obtained as shown in Figure 4c, and then the arrival time of FP can be precisely estimated. A great entropy decrease ocurrs when the signal arrives. Thus the arrival time of the FP can be determined by the decision of the sample which is followed by the great entropy decrease. In consideration of the simplicity and easiness to implement, the turning point of entropy series, as the red point shown in Figure 4c, is determined as the arrival time of the FP. In the following part, the performance of the proposed method will be evaluated with Monte Carlo simulations.

### 3.3. Ranging Performance and Discussion

In this section, the performance of the proposed method will be evaluated with the channel models CM1 to CM4 of the IEEE 802.15.4a standard. The system parameters used in the simulations are set as follows: the Gaussian doublet is selected as the UWB signal, the pulse duration is 0.9 ns, the shape factor is 0.4 ns, center frequency is 2.1 GHz, and band-width is 3.1 GHz. The frame time is 200 ns and the guard interval time is 66.7 ns, *N =* 200, *K =* 6000, and the SNR ranges from −10 to 20 dB with a step of 1 dB. RMSE is also used to evaluate the statistical performance of the proposed method and those of conventional methods, and the parameter of RMSE is set as 100 in the simulation. All the methods are evaluated on the basis of the same parameters.

**Figure 5:**
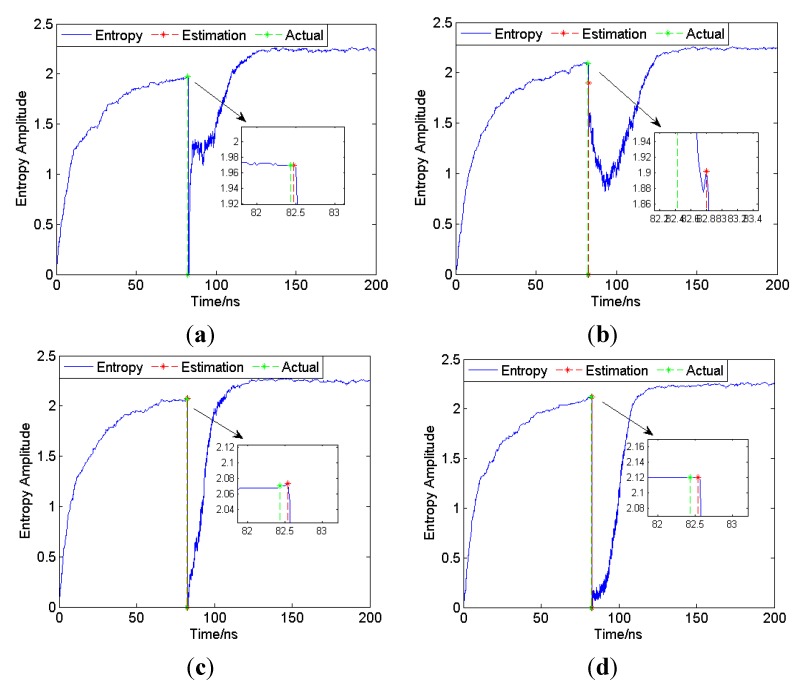
Entropy of received signal of CM1 to CM4 (*SNR* = 10 dB). (**a**) CM1; (**b**) CM2; (**c**) CM3; (**d**) CM4.

First, *SNR* = 10 dB is set as an example to show that the proposed method is generally applicable under all channel conditions. As the results shown in Figure 5 indicate, the entropies of received signals in the CM1 to CM4 channel show a great decrease when the signals arrive, so the proposed entropy-based method is generally applicable. In each subgraph of Figure 5, the details are zoomed in so that the determinations of the FP are clearly visible. Thought the entropy starts to decrease upon the actual arrival of the FP, there is still a small delay between the actual FP and entropy-based estimation, which means the ranging estimation is always positively biased.

**Figure 6:**
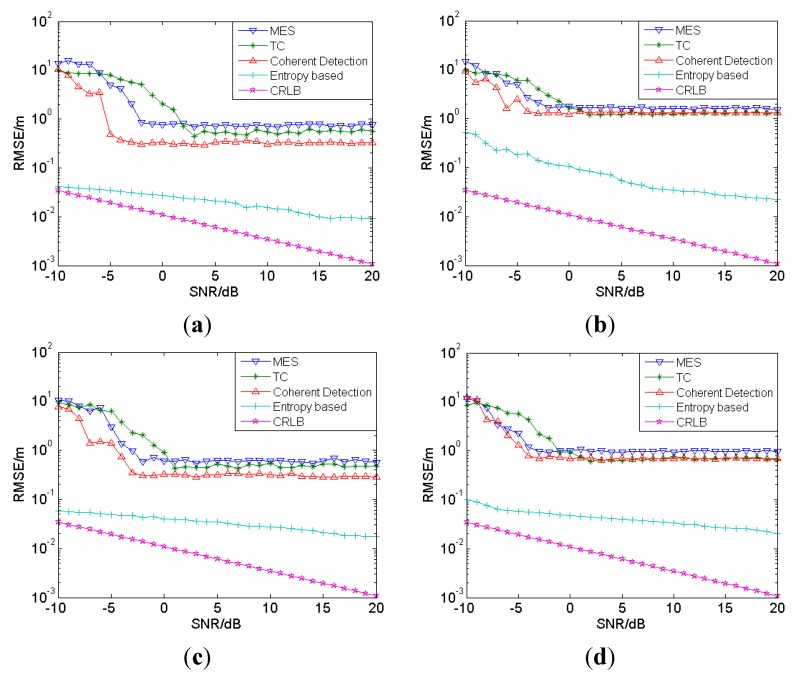
Comparison of proposed method and conventional methods in CM1 to CM4, −10–20 dB. (**a**) CM1; (**b**) CM2; (**c**) CM3; (**d**) CM4.

Then, as shown in Figure 6, a comparison is made of the proposed method and the conventional methods (including MES, normalized TC and coherent detection) in CM1 to CM4, respectively, with SNRs ranging from −10 to 20 dB. According to the literature [12,13] and the simulations done before, the parameters of the conventional methods used in the simulations are set as follows: both MES and normalized TC method are based on the energy of bins and the width of the energy bin is set as 4 ns. MES selects the maximal energy bin and choose the center point of the bin as the arrival time. For normalized TC, the energy bin which first exceeds the threshold is selected and its center point is taken as the arrival time. Normalized TC is a dynamic threshold-crossing method which can adjust the threshold adaptively according to the normalized coefficient $\theta_{norm}$:

(20) $$\theta_{norm}=\frac{\theta -\min\{E_{n}\}}{\max\{E_{n}\}-\min\{E_{n}\}}$$

where θ is the threshold and $E_{n}$ is the energy value of *n*th energy bin. $\theta_{norm}$ is fixed to be 0.6 in the simulation and the corresponding threshold θ can be derived from Equation (20). The coherent estimator is performed by the calculation of the correlation between the received signal and the transmitted Gaussian doublet template, and then by the decision of the output peak as the arrival time.

It can be seen that the performance of the proposed method is better than that of any other conventional method. Under LOS conditions, the RMSE of the ranging error with the proposed method is about several centimeters, and though a little larger, under NLOS conditions, it is still much better than those with other approaches, but there is still a big gap in comparison with Cramer-Rao Lower Bound, especially under NLOS conditions (the purple line in Figure 6). It can be noticed that MES and TC estimation have an error floor, which is due to the time resolution of the energy bin. The error floor exhibited by the coherent estimator is due to the template mismatching, that is, due to the dense multipath and partial overlap of unresolvable pulses of the channel model.

As ranging results are generally used in localization, the accuracy of localization is highly determined by the accuracy of the ranging result. For example, in three-dimensional localizations, at least four anchors are needed, which means that four ranging results between anchors and target node are obtained. If the ranging results are not accurate enough, there will be, when positioning, an accumulation of error which will severely degrade the localization performance. As can be seen in Figure 6, the range errors under NLOS conditions (CM2 and CM4) are still quite large, usually tens of centimeters in size, which will bring large positioning error in localization. Therefore, ranging error mitigation is of great necessity and it will be discussed in the following part.

## 4. SVM Regression-Based Ranging Error Mitigation

Even with the great performance improvements due to the proposed entropy-based ranging method, the ranging errors are still quite large in size under NLOS conditions. In order to reduce the errors under both LOS and NLOS conditions, the SVM regression is introduced. SVM is known as a robust supervised machine learning method which features high performance and outstanding generalization capability. It can efficiently solve a non-linear problem by using what is called the kernel trick, implicitly mapping their inputs into the high-dimensional feature spaces. By using SVM regression, the ranging error can be mitigated without the recognition of the channel conditions or SNRs, which means that the ranging error under all conditions can be mitigated by one regression model. It is much simpler and more practical than any of the NLOS recognition approaches. First, the theory of regression with support vector machines is introduced.

### 4.1. Regression with Support Vector Machines

In this section, the SVM regression method is introduced to perform the ranging error mitigation. In regression, the goal is to infer an unobservable scalar, which is dependent on a set of observable variables. A SVM is a supervised machine learning technique used for classification and regression. It is assumed that there is a sample set $\left(x_{1},y_{1}\right),\left(x_{2},y_{2}\right)\cdots \cdots \left(x_{l},y_{l}\right),x_{i}\in R^{n},y_{i}\in \text{R},i=1,2,...,l$ which is subject to the probability distribution $P\left(x,y\right)\left(x\in R^{n},y\in \text{R}\right)$.We use f(x,y)=w⋅x+b to estimate *y*, which can be interpreted as a hyper-plane. Set *e* as the error between true value and estimated value, then the *ε*-loss function is introduced, as expressed in Equation (21):

(21) $$L\left(e\right)=\left\{ \begin{array}{ll} 0\; & |e|-\varepsilon <0\\ |e|-\varepsilon & \;otherwise \end{array}\right.$$

**Figure 7:**
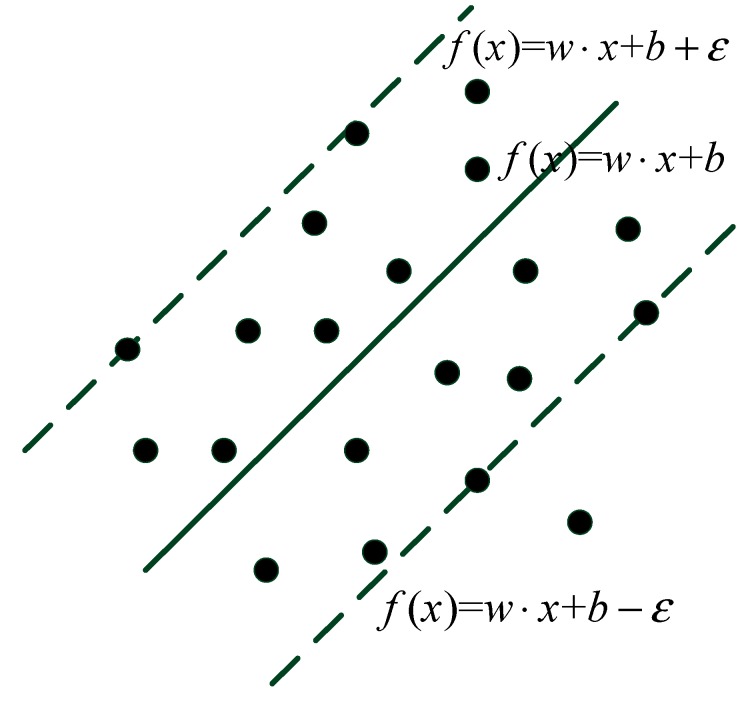
Illustration of ε-range.

Suppose that there exists a hyper-plane which makes L(e)=0, and then there will be two bounding hyper-planes as shown in Figure 7.

The distance between them can be given by Equation (22):

(22) $$d\text{=}2\varepsilon \text{/}\sqrt{{\left\|w\right\|}_{2}^{2}\text{+}1}$$

Hence, the hyper-plane that maximizes the distance between the bounding hyper-planes can be found as Equation (23):

(23) $$\text{minimize}{\left\|w\right\|}_{2}^{2}\;s.t.\;\left\{ \begin{array}{l} y_{k}-w\cdot x_{k}-b\leq \varepsilon \\ y_{k}-w\cdot x_{k}-b\geq -\varepsilon \end{array}\right.$$

In general, when *ε* is too small, the optimization problem becomes infeasible. To make the problem feasible, relaxation factors $\xi_{k},\xi_{k}^{*}$ are introduced and the optimization problem can be written as Equation (24):

(24) $$\text{minimize}{\left\|w\right\|}_{2}^{2}+C\sum_{k=1}^{l}(\xi_{k}+\xi_{k}^{*})\;\;s.t.\;\left\{ \begin{array}{l} y_{k}-w\cdot x_{k}-b\leq \varepsilon +\xi_{k}\\ y_{k}-w\cdot x_{k}-b\geq -\varepsilon -\xi_{k}^{*}\\ \xi_{k}\geq 0\\ \xi_{k}^{*}\geq 0 \end{array}\right.$$

where constant *C* > 0, controls the trade-off between minimizing errors and model complexity. In consideration of the simplicity, a Lagrangian can be introduced to Equation (24), and then, Equation (25) can be obtained:

(25) $$\begin{array}{l} \max W(\alpha )=-\frac{1}{2}\sum_{i,j=1}^{l}\left(\alpha_{i}-\alpha_{i}^{*})(\alpha_{j}-\alpha_{j}^{*})(x_{i}\cdot x_{j}\right)\\ \;+\sum_{i=1}^{l}\alpha_{i}^{*}(y_{i}-\varepsilon )-\sum_{i=1}^{l}\alpha_{i}(y_{i}+\varepsilon )\\ s.t.\left\{ \begin{array}{l} \sum_{i=1}^{l}\left(\alpha_{i}-\alpha_{i}^{*}\right)=0\\ \alpha_{i},\alpha_{i}^{*}\in [0,C/l] \end{array}\right. \end{array}$$

From Equation (25), $w=\sum_{i=1}^{l}(\alpha_{i}-\alpha_{i}^{*})x_{i}$ can be worked out, and then, *f*(*x*) can be given by Equation (26):

(26) $$f(x)=\sum_{i=1}^{n}\left(\alpha_{i}-\alpha_{i}^{*}\right)\left(x\cdot x_{i}\right)+b$$

In the case of the input vector **X** is non-linear in the original space, it should be transformed into a N-dimensional feature vector spaces through a choice of a N-dimensional vector function $\phi :R^{n}\to R^{N}$. In consideration of the decision function in Equation (26) contains the inner-product $\left(x\cdot x_{i}\right)$, the inner-product in N-dimensional space can be expressed as $\left(\phi \left(x_{i}\right)\cdot \phi \left(x_{j}\right)\right)$, and $\left(\phi \left(x_{i}\right)\cdot \phi \left(x_{j}\right)\right)$ ought to be calculated instead of being calculated respectively as $\phi \left(x_{i}\right)$ and $\phi \left(x_{j}\right)$. It is assumed that when there is a function $K\left(x_{i}\cdot x_{j}\right)=\left(\phi \left(x_{i}\right)\cdot \phi \left(x_{j}\right)\right)$, which can transform the inner-product in N-dimensional space into the original space, the problem will be solved. Fortunately this kind of function does exist, known as the kernel function which used to calculate the inner-product in high dimension spaces, and the decision function can be written as:

(27) $$f(x)=\sum_{i=1}^{n}\left(\alpha_{i}-\alpha_{i}^{*}\right)K\left(x\cdot x_{i}\right)+b$$

Given a test vector ***x***, the estimation of *y* can now be obtained. If some features can be extracted as the input vector ***x***, the error can be estimated by calculating *y*.

### 4.2. Feature Selection and Mitigation Procedure

In this part, a description will be given on the feature extraction and on how the SVM regression can be used to perform ranging error mitigation with these features. The features will serve as the input vector *x*, while the ranging error will be the output *y*. First, the details of feature selection will be investigated.

As we can see in Figure 6, when other conditions are equal, the ranging error under LOS conditions is much smaller than that under NLOS conditions. When other conditions are fixed, the ranging error varies with SNR. Thus, the features being selected should be equipped with the capability of distinguishing between LOS and NLOS conditions, as well as the capability of SNR estimation. Meanwhile, under the LOS condition, the strongest path almost corresponds to the first path, but under the NLOS condition, some weak multipath components precede the strongest path. So mean excess delay and root-mean-square (RMS) delay spread can be selected as features because they can describe the degree of multipath of the channel. Some features considered in reference [32] are also included. Taking all these into account, the features extracted can be summarized as shown in Table 2.

**Table 2:** Features extracted.

Name	Expression
Maximum Amplitude	$R_{\max}=\max\left|r(n)\right|$
Energy	$E_{r}=\sum_{n}{\left|r(n)\right|}^{2}$
Mean Excess Delay	$\tau_{m}=\frac{\sum_{l}a_{l}^{2}\tau_{l}}{\sum_{l}a_{l}^{2}}$
RMS Delay Spread	$\tau_{RMS}=\sqrt{\frac{\sum_{k}a_{k}^{2}{\left(\tau_{k}-\tau_{m}\right)}^{2}}{\sum_{k}a_{k}^{2}}}$
Kurtosis	$K=\frac{E(r{\left(n\right)}^{4})}{E^{2}(r{\left(n\right)}^{2})}$ $K=\frac{E(r{\left(n\right)}^{4})}{E^{2}(r{\left(n\right)}^{2})}$
Number of Significant Paths 1 (paths within X dB from the peak)	$\begin{array}{l} N_{1}=\sum_{n}\mathrm{sgn}\left(\left|r(n)\right|>threshold\right)\;\\ \left(threshold=10^{\frac{XdB}{20}}\max\left(\left|r(n)\right|\right)\right) \end{array}$
Number of Significant Paths 2 (captures x% of energy in channel)	$\begin{array}{l} N_{2}=Index\left(\min\left(E_{ce}(n)>x\%\times E_{r}\right)\right)\\ E_{ce}(n)\;is\;cumulative\;energy\;of\;r(n) \end{array}$
Estimated Distance	$\hat{d}$

**Figure 8:**
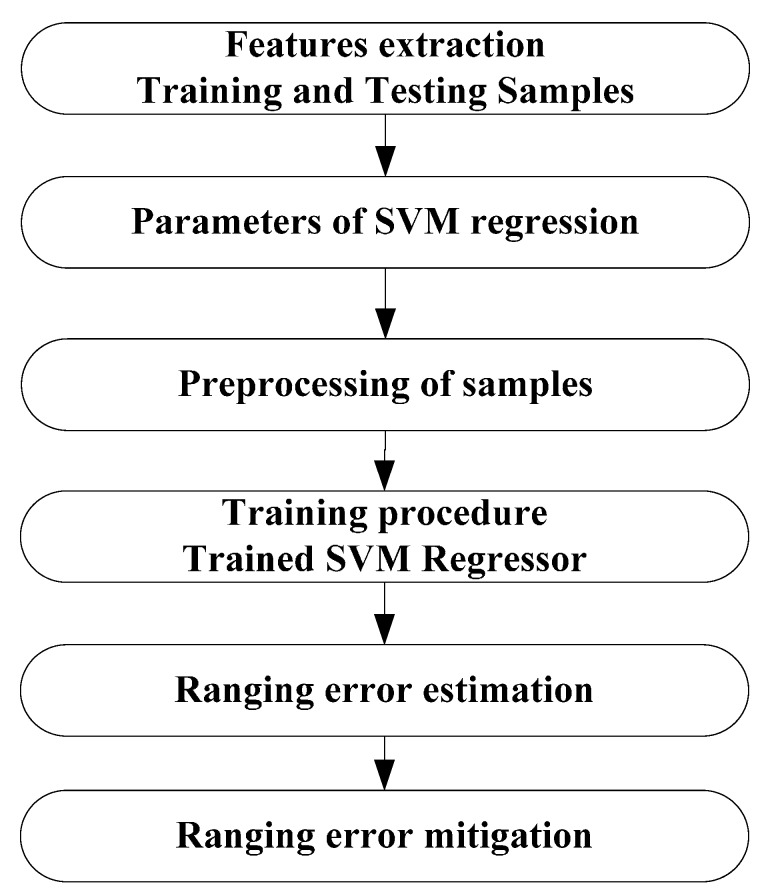
Flow chart of SVM regression.

After the extraction of features from received signals, the error mitigation procedure can be performed, as the flow chart in Figure 8 shows. The database ***S*** consists of training samples. Every training sample is a vector consisting of eight elements (the features), as described in Table 2, along with the corresponding ranging error. Input the database ***S*** into the SVM, and then a trained SVM regressor will be obtained. Another database ***S1*** consisting of testing samples will be inputted into the trained SVM regressor. After that, the ranging error estimation $\hat{\Delta }$ is obtained, which can be used in mitigation. As mentioned above, the ranging estimate is positively biased, so the mitigation procedure can be simply performed by $\hat{d}-\hat{\Delta }$.

### 4.3. Mitigation Performance and Discussion

**Figure 9:**
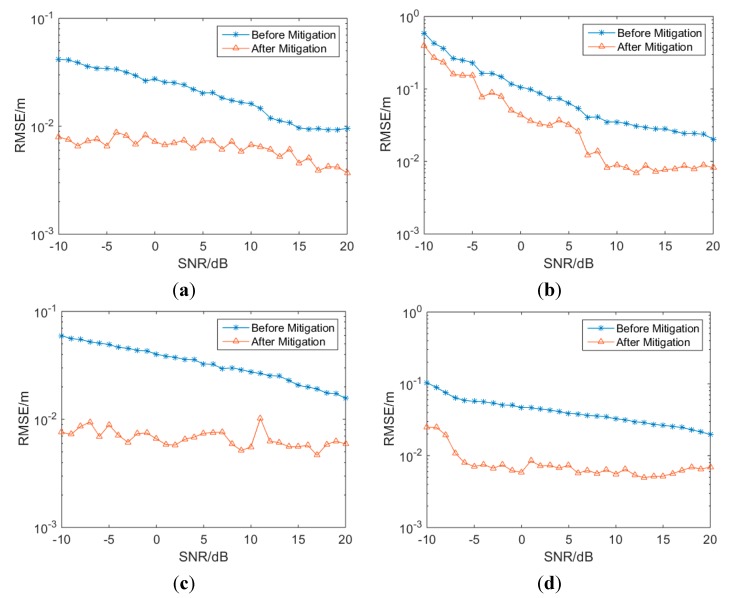
Mitigation results of CM1 to CM4 with SVM. (**a**) CM1; (**b**) CM2; (**c**) CM3; (**d**) CM4.

**Figure 10:**
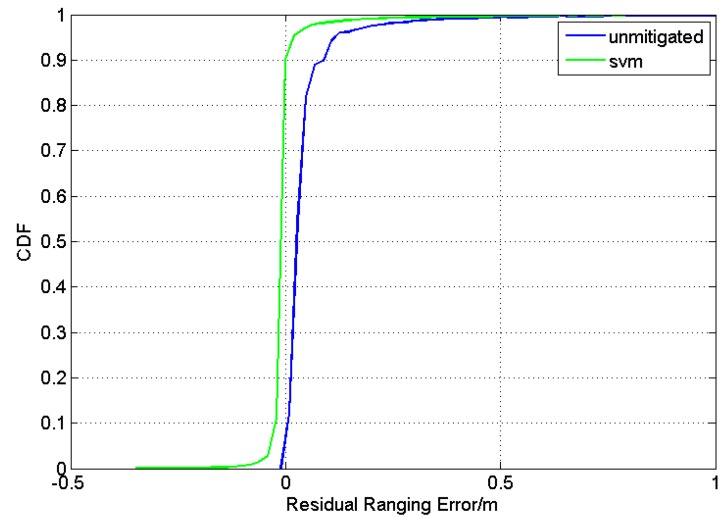
CDF of residual ranging error without mitigation, and using SVM based mitigation.

In this section, the performance of the proposed mitigation method will be evaluated with the channel models CM1 to CM4 in IEEE 802.15.4a. The parameters used in the simulations are set as follows: the LIBSVM [38] package is used in the simulation to achieve the SVM regression. The training dataset ***S*** consists of 12,400 samples (SNRs range from −10 dB to 20 dB with a step of 1 dB, 100 realizations under every channel with every SNR, 12,400 = 100 × 31 × 4), and each sample is a vector consisting of eight features along with the corresponding ranging error. The testing dataset ***S1*** consists of another 12,400 samples and the predicting ranging error $\hat{\Delta }$ will be obtained from the trained SVM regressor. *ε* in loss-function is set as 0.003. Radial Basis Function (RBF) $K(x_{i},x_{j})=\exp\left(-\gamma {\left\|x_{i}-x_{j}\right\|}_{2}^{2}\right)$ is used as the kernel function and γ is set as 2. For numerical reasons, the inputs are converted to the logarithmic domain prior to training. The output of the mitigation procedure is the predicting ranging error $\hat{\Delta }$. The mitigation results and the CDF of the residual ranging error are shown as Figure 9 and Figure 10.

As given in Figure 9, it can be seen that after mitigation, the RMSE decreases considerably in all channels with SNRs ranging from −10 to 20 dB. Before mitigation, the ranging errors are about ten or tens of centimeters, and the ranging errors can be reduced to centimeter-size in most cases after mitigation.

In Figure 10, the CDFs of the residual ranging error before mitigation and after SVM-based mitigation are shown. It can be seen that the green line is much closer to the zero-line of the residual ranging error and much steeper, which means that there are great improvements in the mitigation of ranging errors without the recognition of channel conditions. It is noticed that the residual ranging errors after mitigation can be negative as they are defined as $\hat{d}-d_{1}-\hat{\Delta }$, where $d_{1}$ is the true distance and $\hat{\Delta }$ is the predicted ranging error output by the SVM regression.

## 5. Conclusions

There are a number of problems in conventional approaches when dealing with ranging and ranging-error mitigation in dense multipath and NLOS environments—which in turn cause large-sized errors in the ranging estimation.

The novel approach of entropy-based TOA estimation and SVM-based ranging error mitigation proposed in this paper represents a departure from conventional approaches and leads to great performance improvements, as well as a reduction in the complexity of calculations. The entropy is used to measure the randomness of the received signals and the FP can be determined by the decision of the sample which is followed by a great entropy decrease. The SVM regression is employed to mitigate the ranging errors by the construction of a regression model between ranging error and the features extracted from received signals instead of the recognition of the channel conditions.

Simulation results with the channel models CM1 to CM4 of the IEEE802.15.4a standard have demonstrated the great performance improvements and the robustness of the proposed method. They have also revealed the potential to significantly improve the localization performance in dense multipath indoor environments, which is the target of investigation for our future research.

## Figures and Tables

**Figure A1:**
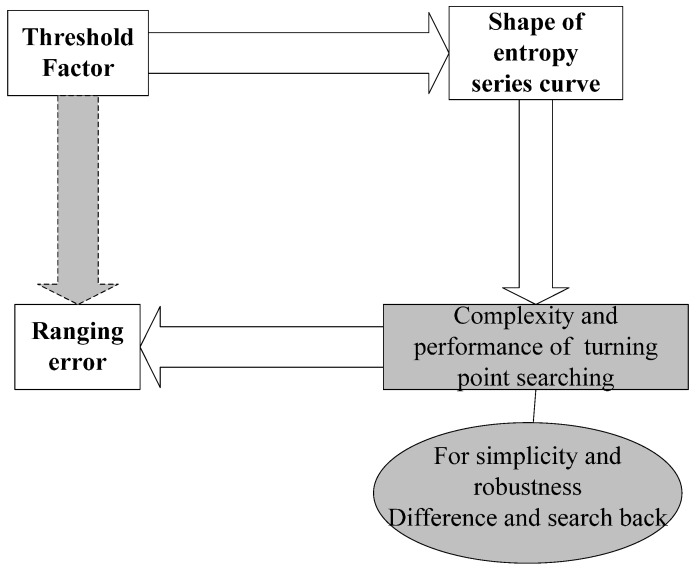
Connection between threshold factor and ranging error.

**Figure A2:**
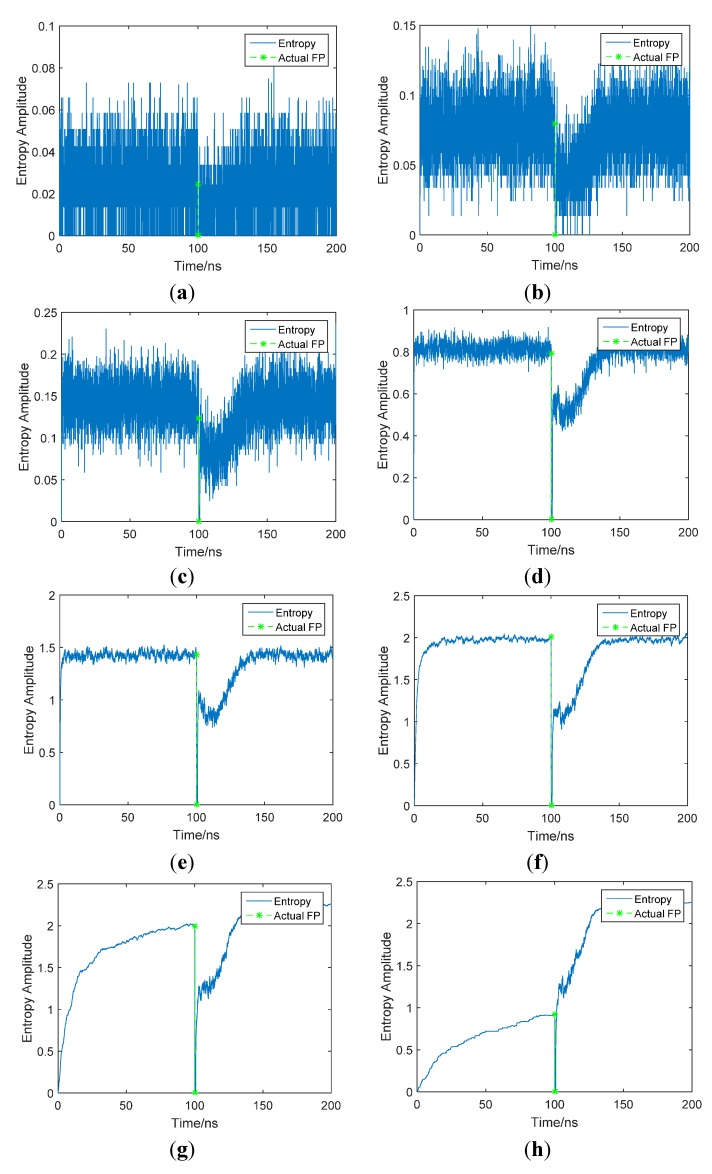

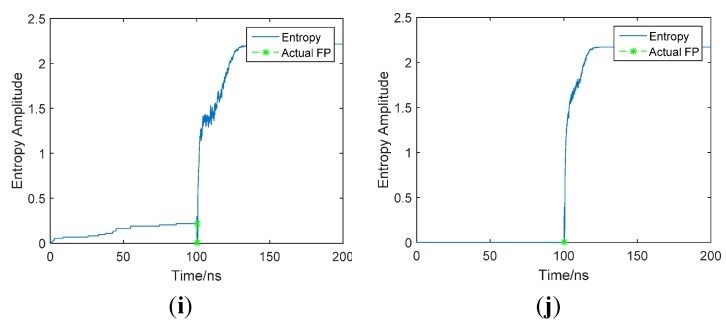
Shape of the entropy series curve with various threshold factors (CM1, SNR = 10 dB) Threshold factor (**a**) 0.1; (**b**) 0.2; (**c**) 0.3; (**d**) 1; (**e**)1.5; (**f**) 2; (**g**) 2.5; (**h**) 3; (**i**) 3.5; (**j**) 10.

**Figure A3:**
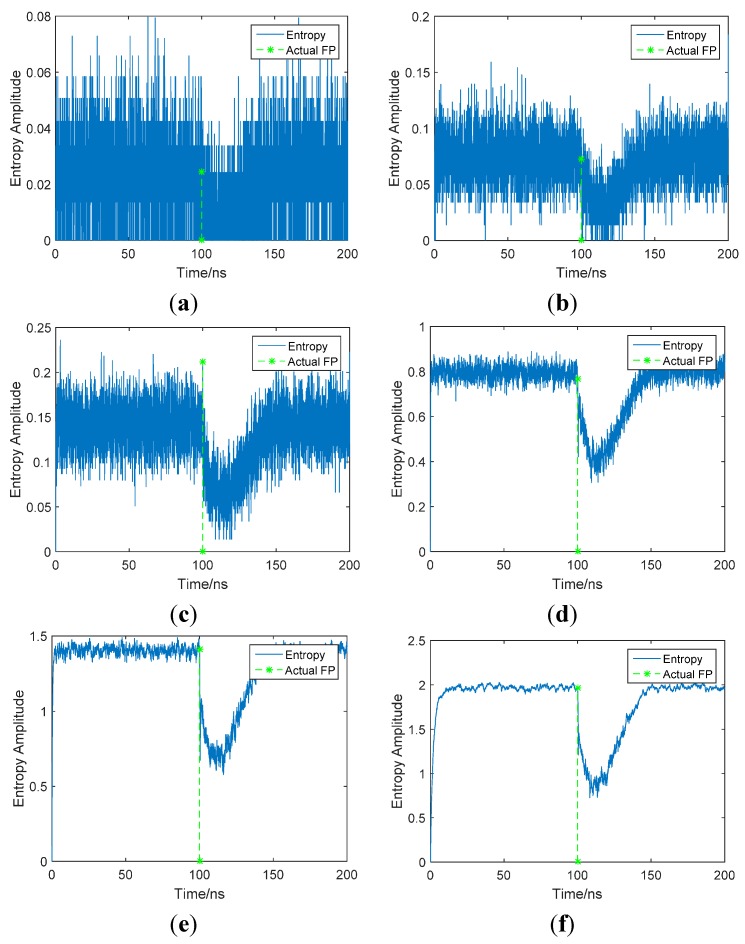

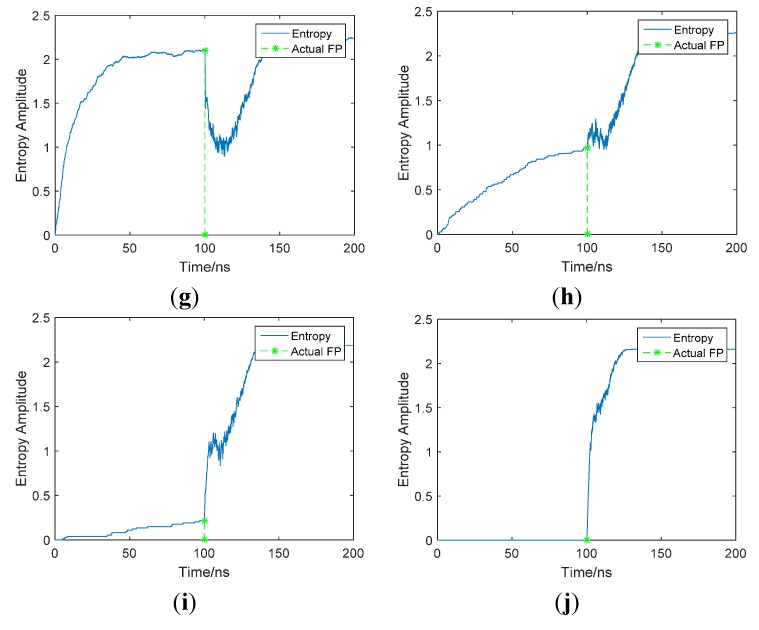
Shape of entropy series curve with various threshold factors (CM2, SNR = 10 dB) Threshold factor (**a**) 0.1; (**b**) 0.2; (**c**)0.3; (**d**) 1; (**e**)1.5; (**f**)2; (**g**) 2.5; (**h**) 3; (**i**) 3.5; (**j**) 10.

## Appendix

### Further Discussion of Threshold Selection in Entropy-Based TOA Estimation

There is no direct connection between threshold factor and ranging error, but, as seen in Figure 3, the ranging error is relatively low at some specific threshold factor values. In order to explain this phenomenon, first, the connection between the threshold factor and the ranging error is investigated, as shown in Figure A1.

The connection between the threshold factor and the ranging error can be described as follows: firstly, the shape and volatility of the entropy series curve is determined by the threshold factor, which can be seen in the following simulation results. Secondly, the complexity and performance of the turning-point searching algorithm are determined by the shape of the entropy series curve. When the entropy-based method is used, the FP can be determined by the decision of the sample which is followed by great entropy decrease, if the entropy curve fluctuates wildly, turning-point searching algorithms of high complexity are needed for the accurate search of the turning point. Finally, ranging errors are determined by the complexity and performance of turning-point searching algorithm. According to these analysis and Figure A1, the indirect connection between threshold factor and ranging error is built which means ranging errors are indirectly determined by the threshold factor.

In our research, for the purpose of simplicity and robustness, the turning-point searching algorithm is fixed to be difference and search-back, so the specific turning-point searching algorithm has a demand for the specific shape of entropy series curve, and the specific shape of entropy series curve has a demand for the threshold factor, which means the ranging error has an indirect demand for the threshold factor.

Figure A2 and Figure A3 are examples of the shape of the entropy series curve with various threshold factors under CM1 and CM2 channel conditions, respectively, when the SNR is 10 dB. As shown in the simulation results, when the threshold factor is relatively small, usually less than 1, the entropy series curve fluctuate wildly, even if the curve decreases when the FP arrives, but it is not clear and hard to determine. The entropy series curves become smoother and the entropy decrease becomes clearer with the threshold factor increasing, and when the threshold factor is around 2.5, the entropy series curve is smooth and the entropy decrease is obvious, and it's easy to determine the turning point by our algorithm. After that, the first half of the entropy series curve (before FP arrival) decreases with the increasing threshold factor, which is caused by rare threshold crossing of noise samples with a large threshold. It is also disadvantageous for the search of the turning point. So it can be seen that 2.5 is a proper value for the threshold factor, as it corresponds desirably with the simulation results in Figure 3.
